# Motor Cortical Networks for Skilled Movements Have Dynamic Properties That Are Related to Accurate Reaching

**DOI:** 10.1155/2011/413543

**Published:** 2011-10-09

**Authors:** David F. Putrino, Zhe Chen, Soumya Ghosh, Emery N. Brown

**Affiliations:** ^1^Neuroscience Statistics Research Laboratory, Massachusetts General Hospital, Harvard Medical School, Boston, MA 02114, USA; ^2^Department of Brain and Cognitive Science, Massachusetts Institute of Technology, Cambridge, MA 02139, USA; ^3^Centre for Neuromuscular and Neurological Disorders, University of Western Australia, QEII Medical Centre, Nedlands, WA 6009, Australia; ^4^Neuroscience Statistics Research Laboratory, Department of Anesthesia, Critical Care and Pain Medicine, Massachusetts General Hospital, 55 Fruit Street, Boston, MA 02114, USA

## Abstract

Neurons in the Primary Motor Cortex (MI) are known to form functional ensembles with one another in order to produce voluntary movement. Neural network changes during skill learning are thought to be involved in improved fluency and accuracy of motor tasks. Unforced errors during skilled tasks provide an avenue to study network connections related to motor learning. In order to investigate network activity in MI, microwires were implanted in the MI of cats trained to perform a reaching task. Spike trains from eight groups of simultaneously recorded cells (95 neurons in total) were acquired. A point process generalized linear model (GLM) was developed to assess simultaneously recorded cells for functional connectivity during reaching attempts where unforced errors or no errors were made. Whilst the same groups of neurons were often functionally connected regardless of trial success, functional connectivity between neurons was significantly different at fine time scales when the outcome of task performance changed. Furthermore, connections were shown to be significantly more robust across multiple latencies during successful trials of task performance. The results of this study indicate that reach-related neurons in MI form dynamic spiking dependencies whose temporal features are highly sensitive to unforced movement errors.

## 1. Introduction

The primary motor cortex (MI) is a cortical region that is responsible for encoding a large repertoire of voluntary movements [1], and the specific role of MI neurons in the production of reaching movements is currently a topic of detailed investigation [2]. During the production of skilled voluntary movements such as reaching, MI is thought to organize into networks of task-related neurons that mediate the production of the motor task. It has been proposed that two or more neurons displaying statistically significant covariation in their spiking patterns, sometimes referred to as a “spiking association”, constitute evidence of network activation [3]. Whilst the presence of significant spiking associations between two neurons, does not necessarily indicate a direct anatomical connection, it does suggest that some form of direct, reciprocal, or common connection may be influencing the firing of both neurons and is referred to as a “functional connection” instead [3]. It is thought that functional connections between MI neurons may allow neurons sharing common involvement in a movement to link together different elements of a task in order to produce the desired movement [4–6]. The incidence of significant spiking associations between reach-related neurons has been shown to increase dramatically with the onset of the reaching task, as compared with periods of postural maintenance [7–9]. 

There is emerging evidence to suggest that the prevalence of spiking associations in MI may also be influenced by the morphological properties of the cells involved. A recent study has shown that during reaching, fast spiking (FS) neurons were significantly more likely to display task-related spiking associations than regular spiking (RS) neurons in MI [10]. Furthermore, inhibitory interneurons in MI have been shown to interact with pyramidal cells in order to appropriately shape their patterns of task-related firing during reaching [11, 12]. This may suggest that the temporal features of neural circuits in MI may influence pyramidal cell output and, thus, task performance. 

Unforced errors during a skilled reaching task may be related to changes in neural activity and interactions occurring in the motor cortex. Comparison of network interactions during errors and those without provides an avenue to study critical variables in neural interactions. Here, we observe unforced errors occurring in a natural (and unpredictable) manner during multiple trials of a learned reaching task. Within a statistical framework based on the point process generalized linear model (GLM), we evaluate the differences in functional connectivity between simultaneously recorded groups of MI neurons when the outcome of task performance varies.

## 2. Materials and Methods

### 2.1. Recording Neuronal Data

The experiments were approved by the Animal Ethics Committee of the University of Western Australia, and the NH&MRC (National Health and Medical Research Council of Australia) guidelines for the use of animal in experiments were followed throughout. Methods of task training and performance, implantation of microwires in the motor cortex, and recording spike activity have been detailed in previous studies [7, 8]. Briefly, two adult cats were trained to perform reaching and withdrawal movements using either forelimb to retrieve food pellets placed between 2 upright Perspex barriers spaced 4 cm apart. The animals were trained in task performance for several months prior to the implantation of the microwires, until the ratio of successful to unsuccessful trials plateaued for at least one month. Only after this level of stabilisation in performance was observed, were the animals considered prepared for recording. The ratio of successful to unsuccessful trials remained stable across the duration of the experiments in each animal, further confirming that motor learning was not taking place during the experiments. Following the completion of behavioural training, general anaesthesia was induced by intramuscular injections of ketamine (6.6 mg/kg), xylazine (0.66 mg/kg), and pentobarbitone (15 mg/kg) and maintained with half the above doses of ketamine, xylazine, and pentobarbitone per hour. PTFE-coated Platinum-Iridium microwires (0.025 mm in diameter, California Wire Company, USA, impedance 0.5–1 MΩ at 1000 Hz) were implanted into the cortex to a depth of about 1.5 mm in forelimb or hindlimb representations of MI (identified by intracortical microstimulation, ICMS). A 32-channel amplifier (X100, PGA32 amplifier, Multichannel Systems, Germany) and several 8-channel preamplifiers (MPA8-1, Multichannel Systems, Germany) were used to record neural activity from up to 24 microwires simultaneously on a computer using MC card and MC rack with a filtering frequency band of 200 Hz–10 kHz (Multichannel Systems, Germany). Activity in each channel was digitized at 25 kHz (sample interval 40 *μ*s). An analogue trigger was concurrently recorded with the neural data and was used to separate periods of neural activity occurring during task performance from periods of continuous raw data acquisition. Analogue trigger signals occurred when the animal's reaching forelimb deformed a laser beam and were used to isolate a 3-second epoch of neural data: starting 1.5 second before and finishing 1.5 second after each trigger signal. The same software also allowed the identification of spike activity in each channel using threshold detectors, window discriminators, and spike waveform analysis. Frame-by-frame video analysis was used to identify five different stages of task performance within these 3-second epochs; “background”, “premovement”, “reaching”, “withdraw”, and “feed” (for a detailed description see [7]). After a set of task trials the animal was trained to sit quietly for 3–5 minutes, and spike activity was also recorded during this period (control period). During control periods no food was expected or given, and animals were observed to be alert. The time stamps of spike events in each channel, for each trial were then exported to MATLAB (MathWorks, Natick, Mass, USA).

In this study, each task trial was classified as either “successful” or “unsuccessful” depending on whether or not the animal was able to reach the food pellet and cover it with its paw in one smoothly performed attempt, as determined by video analysis. The reaching task that was chosen for these experiments is known to have a high degree of intrasubject reliability with regard to movement kinematics and time taken for task performance [7, 13–15]. Similarly, the paw movement used to secure the food pellet in this study is a highly refined skill in cats and has been performed in a stereotyped manner on repetition [16, 17]. Our experimental paradigm utilized a Faraday cage (in order to decrease electrical noise) for all recording sessions. The animals adopted a comfortable posture on entering the cage, and due to limited space, each set of reaching trials was performed with the body in a similar initial posture and orientation. The food pellet was placed in the same starting position by the experimenters across each set of trials, and the trajectory of the animal's paw movements was analyzed in the coronal and horizontal planes using frame-by-frame video analysis in MATLAB (MathWorks, Natick, Mass, USA), in order to monitor the kinematic properties of “successful” and “unsuccessful” attempts at the reaching task. The time stamps of spike activity during task performance were exported to Neuroexplorer software (Nex Technologies, USA), and Peri-Event Time Histograms (PETHs) were used to calculate mean spiking rate during the different stages of task performance in order to identify task-related neurons. Standard ANOVA was used to determine if neural firing rate was significantly modulated during the different stages of task performance compared to “background” periods. In order to be included in this study, isolated neurons were required to show significant firing rate modulation during one or more of the task stages, compared to the baseline firing rate. Thus, only “task-related neurons” were included in this study, and they were recorded from both forelimb and hindlimb regions of MI contralateral to the reaching forelimb.

### 2.2. Histological Location and Classification of Neurons

A description of the experimental data used in our study is summarized in Table 1. In the present study, we have selected simultaneous ensemble neuron recordings from 2 animals and 8 independent datasets. The selection criterion ensured that at least 9 neurons were simultaneously recorded in the datasets that were used. All successful recording sites were found to be located in lamina V of MI (cytoarchitectonic area 4*γ*) upon histological analysis [7].

**Figure 1:**
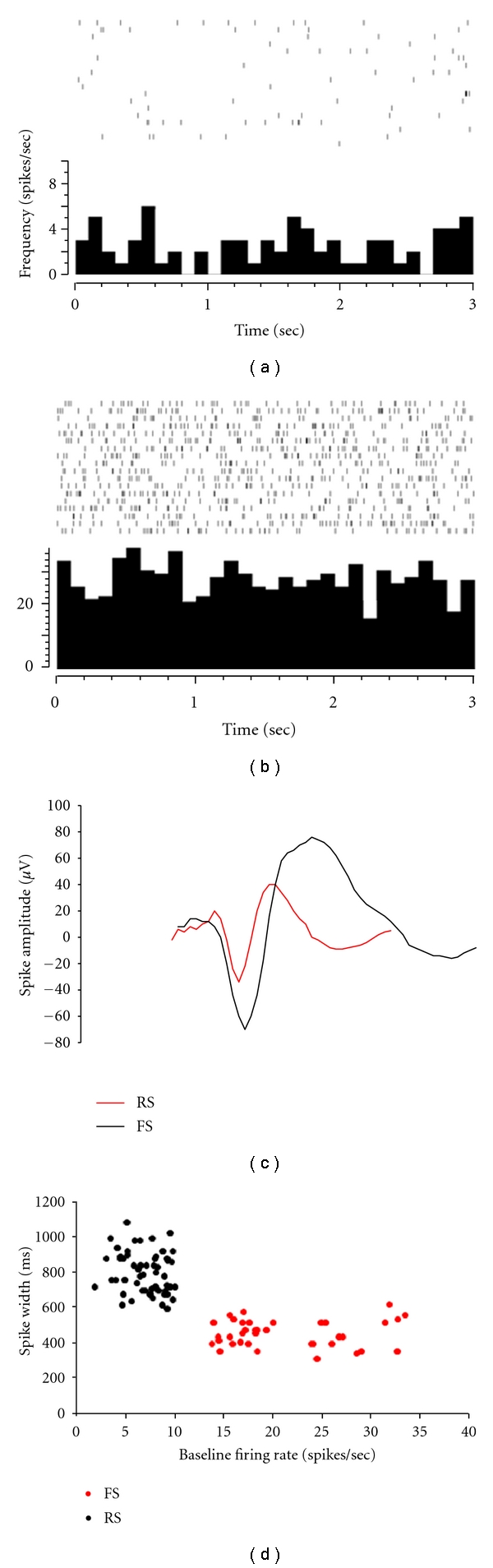
Classification of neurons. Two representative PETHs (bin size 100 ms) illustrating typical neural firing rates during control periods in RS (a) and FS (b) neurons, note the lower mean firing rate in the RS neuron. Spike waveform analysis was also performed on these neurons (c), and differences in spike duration between the averaged waveforms of isolated RS (black) and FS (red) neurons were easily identifiable. When spike duration was plotted against baseline firing rate for all of the neurons recorded from both animals, two distinctly separate clusters emerged (d). Based upon the previously established extracellular criteria, neurons were then classified as either RS (black) or FS (red).

In the past, different studies have successfully classified regularspiking (RS) and fastspiking (FS) neurons based on their baseline firing rates and spike durations [12, 18–21]. With some exceptions, RS and FS cells have been reliably identified as pyramidal cells and inhibitory interneurons, respectively [22–25]. In this study, “baseline firing rate” was defined as the average firing rate of a neuron during control periods (when the animal was sitting quietly) (Figures 1(a) and 1(b)). Analysis of spike duration was performed using the “Spike2” software package (CED, Cambridge, UK), and spike duration was determined using the distance between the two troughs of the action potential (Figure 1(c)), so as to avoid inaccuracies that may arise from difficulties in determining the point of initial deviation from the baseline that has been previously reported [19]. When the information about the baseline firing rate of each of the neurons was added to its corresponding spike duration information and plotted, two clear populations became evident (Figure 1(d)). In the present study, RS neurons were more commonly recorded (58/95) than FS neurons (37/95). Based upon the selection criteria, RS neurons had a baseline firing rate (mean ± SD: 8.5 ± 3.6 spikes/s) that was significantly lower than (*P* < 0.001, rank-sum test) that of the FS neurons (mean ± SD: 22.7 ± 9.6 spikes/s). More recent studies in the primate have also discussed the presence of another class of RS neuron that fires at high frequencies, but still displays the spike duration characteristics of a typical RS neuron [12]. Evidence of this type of cell was not seen in this study, and this may be as a result of a smaller sample size of neurons. 

### 2.3. Point Process Generalized Linear Models

The cross-correlogram and joint peristimulus time histogram (JPSTH) are standard nonparametric methods for analyzing the spiking associations between two neurons [26–28]. However, these nonparametric tools have some drawbacks: first, correlation-based analysis is limited to second-order spike count statistics, which is inadequate for neural spike trains; second, these methods are nonparametric and there is no model validation or goodness-of-fit tests for the data; third, these two methods cannot reveal changes of functional connectivity at fast timescales; fourth, their ability to detect inhibitory spiking associations is limited. To model and quantify the neural interactions among ensemble MI neurons in this study, we used the parametric tool of point process GLM [29, 30]. The point process GLM is a powerful and established statistical tool for modelling extracellularly recorded neural spiking activity, from either single units or networks of isolated cells [30–33]. The main advantage of using a parametric model to characterize neural spiking data is to have a compact model representation of the data and to have the ability to assess data (e.g., variability, comparison between different groups) based on statistical inference (e.g., maximum likelihood estimation, bootstrap analysis) [34, 35]. In the present study, motivated from previous analysis [31], we used the point process GLM to identify the functional connectivity among ensemble neurons during different experimental conditions (background versus reaching, successful versus unsuccessful reaching trials). The computational goal of the study was to make a quantitative comparison between these data using the presented GLM framework. Statistical tests were then used to draw conclusions regarding the significance of our findings for hypothesis testing based on the extracted model statistics [35]. 

Let *c* = 1,…, *C* denote the index of a multivariate (*C*-dimensional) point process. For the *c*th point process (which corresponds to *c*th neuronal firing pattern in the *C*-dimensional neuronal population vector space), let *N*_*c*_(*t*) denote the counting process up to time *t*, and let d*N*_*c*_(*t*) denote the indicator variable, which equals to 1 if there is a spike at time *t* and 0 otherwise. Therefore, multiple neural spike trains are characterized by a multivariate point process d*N*_1:*C*_(0 : *T*). Mathematical background on point process theory can be found in [36].

In modelling the neural spike train point process, the *conditional intensity function* (CIF) is used to characterize the instantaneous firing probability of a spiking event [34, 36]:

(1) $$\lambda_{c}\left(t\;|\;H_{t}\right)=\underset{\Delta \to 0}{\lim}\frac{\text{Pr}\left\{N_{c}\left(t+\Delta \right)-N_{c}\left(t\right)=1\;|\;H_{t}\right\}}{\Delta },$$

where *H*_*t*_ denotes all of ensemble neuronal firing history and any other information (sensory/motor covariates, field potentials) up to time *t*. If the bin size Δ is sufficiently small (in the present experiment we set Δ = 1 ms to assure there is at most 1 spike in each bin), the product *λ*_*c*_(*t* | *H*_*t*_)Δ approximately equates the probability of observing a spike within the interval [*t*, *t* + Δ):

(2) $$\text{Pr}\left\{N_{c}\left(t+\Delta \right)-N_{c}\left(t\right)=1\;|\;H_{t}\right\}\approx \lambda_{c}\left(t\;|\;H_{t}\right)\Delta .$$

Here for simplicity of joint likelihood analysis of ensemble spike trains, we restrict ourselves to the cases where ∑_*c*=1_^*C*^d*N*_*c*_(*t*) ≤ 1 at any time *t* that is, no joint firing is allowed in the continuous-time setting (in the case of discrete-time setting, no joint firing is allowed at the finest temporal scale under consideration). Let *α*_*c*_ denote the unknown parameters in the parametric form of function {*λ*_*c*_}. In the point process GLM framework, we express the CIF in the following log-linear form [31]:

(3) $$\begin{array}{cc} \log\lambda_{c}\left(t\right) & \equiv \log\lambda_{c}\left(t\;|\;\alpha_{c},H_{t}\right)=\alpha_{c}x\left(t\right)=\overset{d}{\underset{j=0}{\sum }}\alpha_{j}^{c}x_{j}\left(t\right)\\ & =\alpha_{0}^{c}+\overset{C}{\underset{i=1}{\sum }}\overset{K}{\underset{k=1}{\sum }}\alpha_{i,k}^{c}x_{i,t-k}, \end{array}\;$$

where dim⁡(*α*_*c*_) = *d* + 1 (where *d* = *C* × *K*) denotes total number of parameters in the augmented parameter vector *α*_*c*_ = {*α*_0_^*c*^, *α*_*i*,*k*_^*c*^}, and *x*(*t*) = {*x*_0_, *x*_*i*,*t*−*k*_}, where *x*_0_ ≡ 1 and *x*_*i*,*t*−*k*_ denotes the spike count from cell *i* at the *k*th time-lag history window. Here, exp⁡(*α*_0_^*c*^) (unit: spikes/s) can be interpreted as the baseline firing rate of neuron *c*. Depending on the algebraic (positive or negative) sign of coefficient *α*_*i*,*k*_^*c*^, exp⁡(*α*_*i*,*k*_^*c*^) can be viewed as a “gain” factor (dimensionless, >1 or <1) that influences the current firing probability of neuron *c* from the spike count fired by another neuron *i* at the previous *k*th time lag. We can further define the mean excitatory-plus-inhibitory (*E* + *I*) ratio, averaged across *K* lags, as

(4) $$\text{ratio}=\frac{1}{K}\overset{K}{\underset{k=1}{\sum }}\overset{C}{\underset{c=1}{\sum }}\overset{C}{\underset{i=1}{\sum }}\frac{\#\left\{\left|\alpha_{i,k}^{c}\right|\gg 0\right\}}{C\left(C-1\right)},$$

where ≫ denotes “significantly greater than 0”. 

Let *θ* = {*α*_1_,…, *α*_*C*_}, where dim⁡(*θ*) = *C*(1 + *d*). By assuming that conditional to spiking history of neuronal populations, the ensemble neuronal spike trains are mutually *conditionally independent *(Note that this is not to be confused with assuming that the spike trains or neurons are mutually statistically independent), the continuous-time log-likelihood of all observed spike train data {d*N*_*c*_(0:*T*)}_*c*=1_^*C*^ is written as [31, 34]

(5) $$\begin{array}{cc} L\left(\theta \right) & =\overset{C}{\underset{c=1}{\sum }}L\left(\alpha_{c}\right)\\ & =\overset{C}{\underset{c=1}{\sum }}\left\{\overset{T}{\underset{0}{\int }}-\lambda_{c}\left(t\;|\;\alpha_{c}\right)\text{d}t+\overset{T}{\underset{0}{\int }}\log\lambda_{c}\left(t\;|\;\alpha_{c}\right)\text{d}N_{c}\left(t\right)\right\}. \end{array}$$

By discretization of (5), we also obtain the discrete-time log-likelihood function, in which the integration will be replaced by a finite sum. From (5) it is clear that—*L*(*θ*) is convex with respect to (w.r.t.) each *λ*_*c*_ as well as *α*_*c*_ (because of the log-linear form in (3)). In addition, the index *c* is uncoupled from each other in the network log-likelihood function, which implies that we can optimize the function separately for each spike train observations d*N*_*c*_(0 : *T*) once the parametric form of *λ*_*c*_(*t*) is specified. For simplicity, we will drop off the index *c* at *λ*_*c*_ and *α*_*c*_ when no confusion occurs.

### 2.4. Regularisation, Goodness of Fit, and Model Selection

To address the “sparse spiking data” problem (which refers to fitting certain cells with a low mean firing rate and small number of trials), we use a penalized maximum likelihood estimation method in order to avoid overfitting [37]. The basic idea of regularization is to impose certain constraints or priors on the parameters, which optimize the out-of-sample performance. Specifically, we aim to maximize following penalized log-likelihood function, denoted by *L*_*p*_(*α*) [32]:

(6) $$L_{p}\left(\alpha \right)=\overset{T}{\underset{0}{\int }}-\lambda \left(t\;|\;\alpha \right)\text{d}t+\overset{T}{\underset{0}{\int }}\log\lambda \left(t\;|\;\alpha \right)dN\left(t\right)-\rho \alpha^{T}Q\alpha ,$$

where *ρ* > 0 denotes a regularization parameter, and **Q** denotes a user-defined, positive semidefinite matrix. Different choices of matrix **Q** lead to different regularization solutions. As a special case, when **Q** = **I** (identity matrix), the standard “ridge regression” is recovered. Further discussion regarding the choice of a smoothing operator for matrix **Q** is detailed in [37]. The regularization coefficient *ρ* was selected by leave-one-out cross-validation, and the optimization can be efficiently implemented by the conjugate gradient method [37]. Since the optimization problem is convex, the final estimate is globally optimal. 

Upon convergence of the optimization algorithm (using the criterion that the iteration stops when the log-likelihood change in two subsequent updates is less than 10^−4^), the goodness of fit of the point process model is evaluated based on the *Time-Rescaling Theorem* and *Kolmogorov-Smirnov* (KS) test [34]. In the model described in (3), we have assumed that the number *K* (i.e., the number of spiking history windows) is defined. In practice, this number is unknown and needs to be determined using a model selection procedure. The major issues faced with model selection relate to the length of past spiking history that is required and the choices of window size and window number. In our experiment, we minimized the *Bayesian information criterion* (BIC) to select the window number. The BIC attempts to find a suboptimal tradeoff between the model size and the log-likelihood:

(7) BIC=−2L(α)+dlog⁡l,

where *d* = dim⁡(*α*) denotes the size of the unknown parameters, and *l* denotes the total number of 0/1 binary samples. BIC is derived by taking the second-order Taylor expansion around the log posterior such that it avoids the model and parameter integration during model selection [38]. Window size is typically chosen in such a way that the most recent windows use a small bin size, followed by gradually increasing bin size (the choice of log scale of window bin size roughly accounts for exponentially decaying dependence on preceding spike counts). The maximum length of spiking history was tested from 30 ms up to 120 ms (since the reaching period only lasted about 700 ms), but in many cases it was found that a shorter history length (e.g., 40–60 ms) is sufficient for achieving a good KS statistic and lower BIC, suggesting that a compact model is preferred in model fitting.

For small or sparse spiking data sets, our simulation studies have confirmed that the regularization imposed on penalized maximum likelihood estimate often significantly improves the goodness of fit of the model (Data not shown; see [39]).

### 2.5. Identifying Functional Connectivity

Functional connectivity between simultaneously recorded neurons shall be defined in this paper as the *statistically significant spiking dependence of one cell on the other*. Upon completing the statistical inference, we obtain the penalized maximum likelihood estimate (MLE) of parameter (denoted by $\hat{\alpha }$ of the point process GLM. Let *E*[·] denote the mathematical expectation operator, and let

(8) $$\begin{array}{cc} \Sigma & =-E{\left[\frac{\partial^{2}L}{\partial \hat{\alpha }\partial {\hat{\alpha }}^{T}}\right]}^{-1}\\ & \approx {\left\{\overset{T/\Delta }{\underset{t=1}{\sum }}\left[\nabla \lambda_{t}\nabla {\lambda_{t}}^{T}\frac{\Delta }{\lambda_{t}}-\frac{\nabla^{2}\lambda_{t}}{\lambda_{t}}\left(\text{d}N(t)-\lambda_{t}\Delta \right)\right]\right\}}^{-1} \end{array}$$

denote the negative Hessian matrix of the log-likelihood estimated from the ensemble samples, where $\nabla \lambda_{t}=\partial \lambda_{t}/\partial \hat{\alpha }$ and $\nabla^{2}\lambda_{t}=\partial^{2}\lambda_{t}/\partial \hat{\alpha }\partial {\hat{\alpha }}^{T}$ denotes the first- and second-order partial derivatives of the CIF with respect to $\hat{\alpha }$ at a discrete-time index *t*, respectively. From the property of MLE it is known that Σ approximates the inverse of the Fisher information matrix; in addition, under the regularity condition and large sample assumption, the MLE asymptotically follows a multivariate Gaussian distribution [34, 38]: $\hat{\alpha }\sim N(\alpha ,\Sigma )$, from which we can further derive the 95% Wald confidence bounds of each element in *α* (i.e., ${\hat{\alpha }}_{i}\pm 1.96\Sigma_{ii}^{1/2}$). Provided that any of the coefficients are significantly different from zero, or their 95% Wald confidence intervals are not overlapping with 0, we conclude that the estimated nonzero coefficients are significant, and the “connection” between two cells at a certain time-lag is either excitatory (positive) or inhibitory (negative). Unlike cross-correlation, the pairwise spiking dependence is directional and asymmetric in our statistical models, and the spiking dependence between A→B and B→A is not necessarily the same. Therefore, for *C* ensemble neurons, there are possibly (*C* ^2^ − *C*) directions (excluding *C* self-connections) between all neuron pairs. 

In summary, our point process GLM network analysis consists of several steps: (i) model selection and model determination based on the penalized maximum likelihood estimation, plus model goodness-of-fit tests, (ii) determination of statistically significant nonzero GLM coefficients (algebraic sign and total number) at different time lags, (iii) repeat the analysis for different experimental conditions, (iv) use of statistical tests to make quantitative comparison between different experimental conditions.

## 3. Results

### 3.1. Evaluation of Errors in Reaching

**Figure 2:**
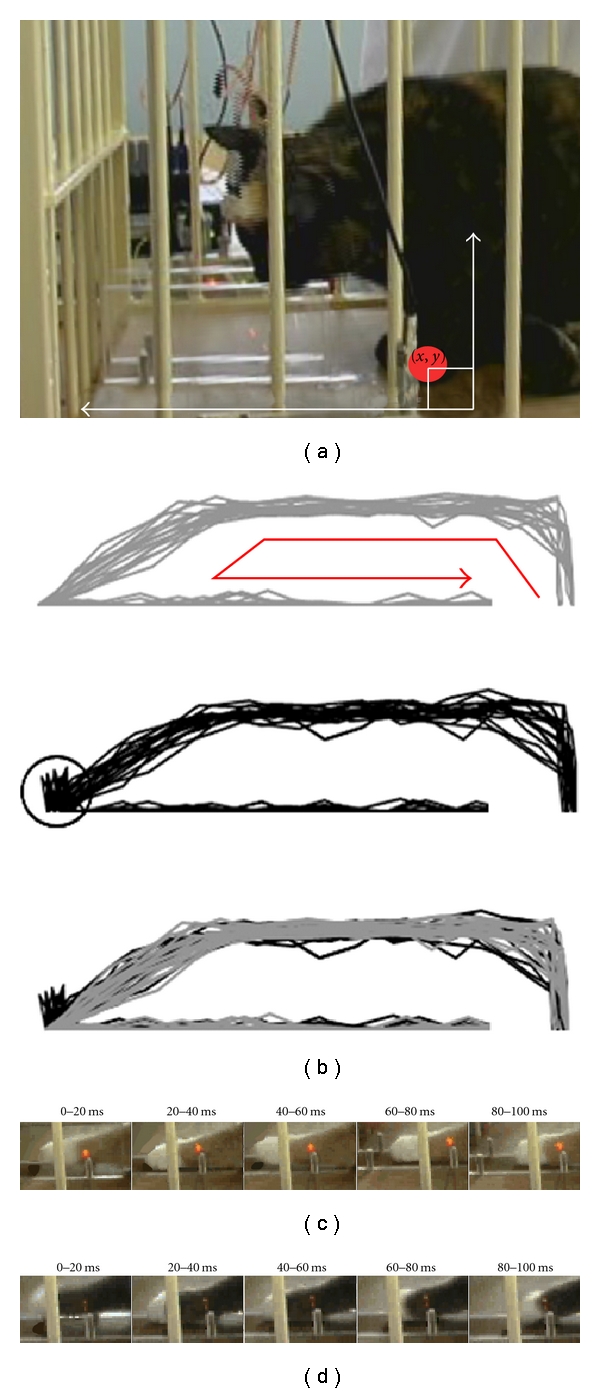
Kinematic analysis of task performance. Frame-by-frame analysis was performed using MATLAB in order to create two dimensional trajectory traces for the reaching movement in the coronal and horizontal planes. The coordinates were derived by locating the central point of the animal's reaching paw at each separate frame of video footage (a). In (b), the results of trajectory analysis for dataset 4 are displayed for successful (top) and unsuccessful (middle) trials. The red arrow inside the successful trajectory trace indicates the direction of the reaching motion, and the final trajectory trace (bottom) is the result of overlaying the successful and unsuccessful traces. Note the clear similarity in the trajectory paths of the successful and unsuccessful trials, but the slight difference at the end of the reach phase of the movement (circled) created by a second (or occasionally third) attempt at grasping the food pellet during unsuccessful trials. Finally, an example of frame-by-frame analysis that was performed at the end of the reaching stage (as the food pellet was secured) that allowed for the classification of trials as either unsuccessful (c) or successful (d). Note the slightly prolonged ending of the reach stage in “(c)” as the animal needs to make another attempt to grasp the food pellet; while at similar frames in “(d)”, the commencement of the withdrawal stage had already begun.

Once fully trained, the amount of time taken for the animals to perform each stage of task performance remained rather stable over several weeks to months before recording began, and this phenomenon has been described in a previous study [7]. A reaching trial was defined as “unsuccessful” if the animal was unable to close its paw over the offered food pellet at the end of the “reach” stage of task performance in a single smooth attempt. Unsuccessful attempts at task performance were clearly evident on video analysis and typically required no more than two extra attempts at securing the pellet. Each rapid correction after an initial unsuccessful attempt involved abduction and retraction of the shoulder and flexion of the elbow in order to lift the forelimb off the ground, followed by an obligatory protraction and adduction of the shoulder and extension of the elbow in order to repeat the terminal period of the “reach” stage of task performance. A trace of the animals' paw trajectory during task performance was created (Figures 2(a) and 2(b)) in order to assess any major differences in limb trajectory that may have occurred leading up to the error in securing the food pellet. No major difference in the trajectory traces of successful and unsuccessful trials could be detected in the trajectory traces of either animal as they reached towards the food pellet. However, the rapid movement corrections that occurred following unsuccessful attempts at securing the pellet were clearly distinguishable from the trajectory analysis (circled region in Figure 2(b)). Corrections to the movement pattern were implemented rather quickly, with another grasping attempt completed within 40–60 ms of the preceding one (Figures 2(c) and 2(d)); A statistically significant increase in the length of time taken to perform the “reach” stage of task performance was observed during unsuccessful trials in both animals (*P* < 0.05, Student's *t*-test; Table 2). A slight decrease was also observed in the length of time taken to perform the withdrawal stage of task performance during unsuccessful trials in both animals, but this trend was not seen to be significant. Animal 2 was also seen to have a much higher rate of successful task performance than Animal 1, but the recordings used in this study were taken over a period of 1 month, and the animals' success rates remained unchanged throughout. 

### 3.2. Cortical Location of Successfully Recorded Neurons

Upon histological analysis, successful recording sites were all subsequently found to be located in the primary motor area (cytoarchitectonic area 4*γ*; [7]) in the anterior or posterior sigmoid gyri or in the rostral or caudal lip of the cruciate sulcus and in the deeper layers of the cortex (Lamina V). Many of the unsuccessfully implanted microwires were subsequently found to be located in the white matter; no spike activity could be recorded through these electrodes and neither was any movement evoked by ICMS. We also distinguished between recording sites in the rostral (r4*γ*) and caudal (c4*γ*) subdivisions of the primary motor area, separated by the cruciate sulcus, in our analysis [40–43]. In the present study, 95 neurons were identified from successful recording sites in r4*γ* (57/95) and c4*γ* (38/95). The majority of these neurons were recorded from sites that evoked forelimb (59/95) movements during ICMS sessions, and RS (58/95) neurons were more commonly seen than FS (37/95) neurons.

### 3.3. Fitting a Point Process GLM from Spiking Ensemble Neurons

Model-based inference was used to characterise the functional connectivity of ensemble neurons using the point process GLM [29, 30, 44]. Based on our model selection criterion, in Dataset-1 we selected 9 firing history windows (up to preceding 40 ms) that include the spike counts in the past 1~3, 4~6, 7~9, 10~12, 13~15, 16~20, 21~25, 26~30, and 31~40 ms, respectively. In this dataset there are 13 × 9 spiking history windows (i.e., each cell contributes 9 preceding windows of spike counts), and dim⁡(*α*) is 118 for the point process GLM.

Assuming stationary model statistics across trials, unless stated otherwise, all trials in each dataset were used to fit the point process GLM. The selected history windows for the remaining cases were given as follows.

*Dataset 2:* [1~3, 4~6, 7~9, 10~12, 13~15, 16~20, 21~25, 26~30, 31~40, 41~50].
*Dataset 3:* [1~3, 4~6, 7~9, 10~12, 13~15, 16~20, 21~25, 26~30, 31~40].
*Dataset 4:* [1~3, 4~6, 7~9, 10~12, 13~15, 16~20, 21~25, 26~30, 31~40].
*Dataset 5:* [1~3, 4~6, 7~9, 10~12, 13~15, 16~18, 19~21, 22~24, 25~30, 31~40].
*Dataset 6:* [1~3, 4~6, 7~9, 10~12, 13~15, 16~18, 19~21, 22~24, 25~30, 31~40].
*Dataset 7:* [1~3, 4~6, 7~9, 10~12, 13~15, 16~20, 21~25, 26~30, 31~40].
*Dataset 8:* [1~3, 4~6, 7~9, 10~12, 13~15, 16~20, 21~25, 26~30, 31~40].

In each case, an efficient optimisation procedure was applied for inferring the parameters of point process GLM, followed by goodness of fit assessment. Amongst a total of 95 × 4 = 380 model fittings (95 cells, 4 conditions: background/reaching, succ/unsucc), nearly 73% of cases fit very well with our point process GLM using a network likelihood model; that is, their KS plots fell within the 95% confidence intervals. When the confidence bound criterion was relaxed to 90%, the ratio of significance increases to 92%. This suggests that the point process GLM that was selected for use in this analysis characterized our neuronal spike train data rather well. For each cell, the size of unknown parameters in the network likelihood model varies from 118 to 151, which points at a relatively compact parametric model. Cells with less satisfactory fitting results may be as a result of a number of factors, such as: (i) the model is not sufficient to capture a long-range spiking dependence due to its compact size, or (ii) if the target cell's spiking activity is being too heavily influenced by uncontrollable factors such as unrecorded neuronal spiking activity or strong firing modulation in response to motor variables, the model may not be able to predict the cell's spiking as accurately. The latter would appear most likely, since an experimental extension of the spiking history length did not result in any significant improvement in model fitting. Once model goodness-of-fit was established, we may infer and compare statistics extracted from the statistical model [44, 45]. Extracted model-based statistics, once being identified statistically significant, will then be used to characterize the difference between distinct experimental conditions (background versus reaching, succ versus unsucc; Table 3). 

### 3.4. Spiking Associations Specific to Different Cell Group Pairings

In order to examine the incidence of functional connectivity between different cell types during different behavioural conditions, a classification according to neuronal subtype (RS-FS, RS-RS, FS-FS) was made, and incidence of spiking associations within these three groups was computed. Two cells are considered to have a directional interaction if one or more of the GLM coefficients associated with the selected history windows return a statistically significant nonzero result. To make a fair comparison of the incidence of neuronal associations between the different cell pairings, the raw number of significant associations was divided by the number of all possible pairings that were tested for evidence of association to obtain the ratio of neuronal interactions (summarised in Table 3). Analysis of the eight datasets showed that during background periods, spiking associations between FS-FS pairs were the most common, followed by RS-FS and RS-RS pairs. During task periods, spiking associations were still most commonly seen between FS-FS pairs however, RS-RS became the next most commonly seen, followed by RS-FS pairs. 

 To quantify and compare these differences between successful and unsuccessful trials, we conducted the paired rank-sum test. It was found that during the reaching period, the median fraction values between RS-RS and FS-FS (unsucc: *P* < 0.001; succ: *P* < 0.006), RS-FS and FS-FS (unsucc: *P* < 0.001; succ: *P* < 0.006), and RS-RS and RS-FS (unsucc: *P* < 0.006; succ: *P* < 0.006) cell pairings, all reached statistical significance. Thus, while significant differences were seen in the prevalence of spiking associations between different cell groups, these findings were not different when successful and unsuccessful trials were compared.

Spiking associations were more commonly observed during task periods (compared with background periods) in all three of the cell pairings investigated (RS-FS, RS-RS, FS-FS), a finding that was consistent regardless of trial success (Table 3). Furthermore, when the animal's behaviour changed from postural maintenance (background) to task performance, the incidence of significant spiking associations detected in RS-RS, RS-FS, and FS-FS cell pairings increased by 356%, 53%, and 12%, respectively during successful trials, and during unsuccessful trials, these numbers were 346%, 71%, and 22%, respectively. However, these numbers shall be treated carefully since there is a high degree of variability among different data sets. These findings indicate that the onset of a voluntary movement such as target reaching is accompanied by a dramatic increase in the prevalence of neuronal interactions between pairs of putative excitatory neurons. However, despite this proportionately large increase in the number of RS-RS associations, spiking relationships between FS neurons still remain the most commonly seen during reaching.

### 3.5. Factors Affecting the Incidence of Neuronal Interactions

A global quantitative assessment of the results reported in Table 3 was conducted, considering multiple factors all together. We used a multiway ANOVA to test whether the means of several groups are equal. The incidence of functional connectivity between different cell groups (RS-RS, RS-FS, FS-FS), task outcomes (successful versus unsuccessful), and periods (background versus reaching) were considered as three potential factors (denoted by *g*1, *g*2, *g*3, resp.), and those factors were tested (together as well as individually) to see if they reached significance across all datasets. A random-effects three-factor ANOVA revealed that only two factors (cell group and period) reach statistical significance, and the result is summarized in Table 4.

It should be pointed out that although the skill factor did not reach significance in the ANOVA test, it did not rule out the possibility that only a subset of ensemble neurons were responsible for causing errors in the grasping motion. Inclusion of all neurons might make it difficult to reveal the “significance” of performance-related encoding specific to certain cells.

### 3.6. Overall Incidence and Strength of Significant Spiking Associations

Based on the selected model, we can compute the mean connectivity ratio at different time lags to characterize the time-dependent spiking associations detected amongst simultaneously recorded neurons. This ratio was defined as the total number of significant nonzero (positive or negative) coefficients detected against the total number of neuronal pairs assessed across all finite discrete time lags and normalised by the number of time lags and was called the mean (*E* + *I*) connectivity ratio (4). In the previous section, the fraction of detected neuronal interactions was reported across cell groups and behavioural conditions. However, computation of the mean (*E* + *I*) connectivity ratio allows for further insight into the nature of the detected connectivity, as it provides quantitative information about how the strength and incidence of the significant nonzero coefficients vary across multiple time windows.

The mean (*E* + *I*) connectivity ratios in the “background” and “reaching” epochs are summarized for all datasets in Table 3 (third column), and the ratio values vary between 0.07 and 0.86 under different conditions. The mean (*E* + *I*) connectivity ratios were seen to increase significantly during successful (0.54 ± 0.26) compared with unsuccessful (0.37 ± 0.21) trials during task periods, indicating that the incidence of overall spiking association was affected by the success of task performance (*P* < 0.001, paired rank-sum test). During successful trials, the mean (*E* + *I*) connectivity ratio increased significantly during task performance (0.54 ± 0.26) compared with the background period (0.38 ± 0.21) (*P* < 0.001, paired rank sum test). Interestingly, this was not seen during unsuccessful trials, where no significant difference could be detected between the mean (*E* + *I*) connectivity ratio during background (0.33 ± 0.19) and task performance (0.37 ± 0.21). Therefore, while we have shown that the onset of reaching was accompanied with a significant increase in spiking associations between simultaneously recorded neurons during both successful and unsuccessful trials, these data suggest that the spiking associations detected during unsuccessful trials are less robust than those seen during successful trials. 

**Figure 3:**
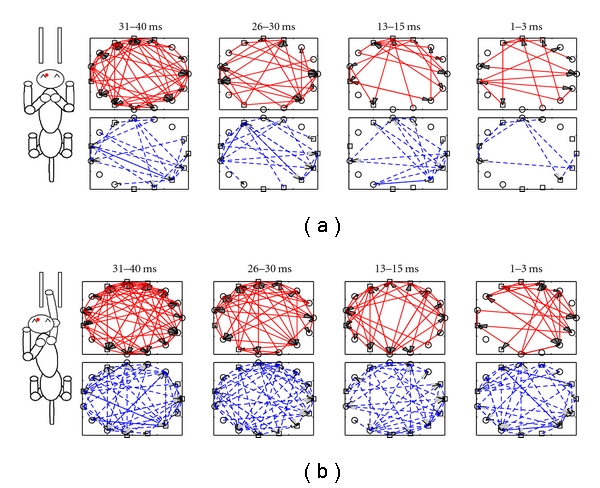
Illustrations of excitatory (red, top row) and inhibitory (blue, bottom row) functional connectivity among 15 ensemble MI neurons (Dataset-3) recorded from the left cerebral hemisphere of one of the animals during the background (a) and reaching (b) stages of successful task trials. Circles and squares represent the RS and FS cells, respectively. Uni- or bidirectional arrow indicates directional statistical dependence between cells, with solid/dashed lines representing excitatory/inhibitory connections. The numbers above each panel of connectivity maps indicate the different spiking history windows that were selected to detect the associations. Note the significant increase in both inhibitory and excitatory spiking associations that occurred during reaching when compared with background, and different pairs of neurons are involved in functional interactions during different behavioural conditions.

Figure 3 allows for the separate observation of excitatory and inhibitory connections as they occur over the different time history windows during background and reaching periods. This particular study uses a novel method of network analysis that allows for the examination of inhibitory spiking associations with unprecedented accuracy. Whilst inhibitory connections are slightly less common than excitatory connections, there is a consistent ratio of excitatory to inhibitory functional connectivity. In order to measure this, an excitatory-to-inhibitory (*E* : *I*) ratio was constructed, which measured the total number of excitatory connections, divided by the number of inhibitory connections detected in each dataset during both the background and reaching conditions. This differs from the mean *E* + *I* connectivity ratio, which measures the *average* number of excitatory *and* inhibitory connections during these conditions. Interestingly, although we showed a significant increase in the mean *E* + *I* connectivity ratio between reaching compared with background periods, the *E* : *I* ratio remains constant throughout background (1.11 ± 0.22) and reaching (1.11 ± 0.11). This suggests that although there is significant increase in the prevalence of functional connections with the onset of reaching, the balance of excitation and inhibition remains the same. Similarly, the *E* : *I* ratio remained stable during successful (1.11 ± 0.11) and unsuccessful trials (1.1 ± 0.19) despite a significant difference in their mean *E* + *I* connectivity ratio, suggesting that this specific *E* : *I* ratio may be a fairly robust ratio that is observed during normal cortical function.

### 3.7. Dynamic Changes in Spiking Associations during Errors in Reaching

When all datasets used in this study were considered, the median values measuring the incidence of significant spiking associations between pairs of simultaneously recorded neurons did not reach statistical significance (*P* > 0.05, rank-sum test) when a comparison was made between the successful and unsuccessful behavioural conditions. 

**Figure 4:**
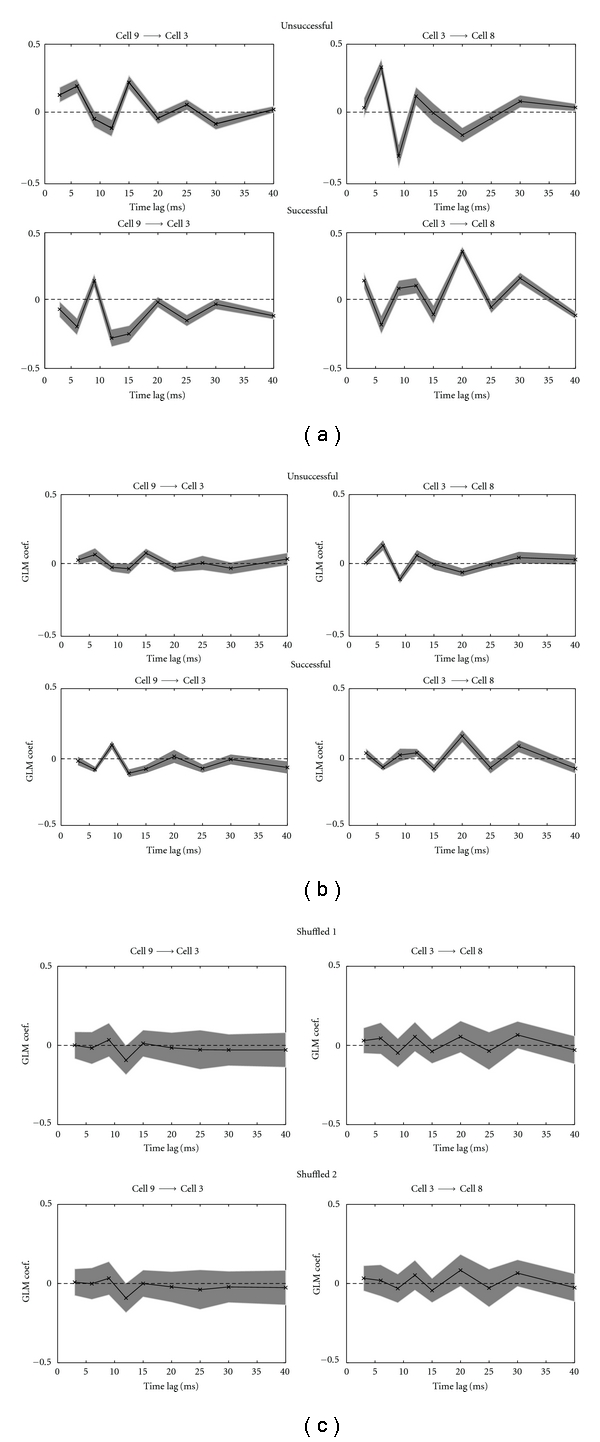
Two representative examples of estimated GLM coefficients {*α*_*i*,*k*_} (shown in vertical axis, dimensionless) of cell pair interactions between unsuccessful and successful trials during reaching movement. Positive/negative coefficients represent excitatory/inhibitory effects. Notation *c* → *d* denotes the directional spiking dependence from trigger cell *c* to target cell *d*. The shaded areas around the curve indicate the 95% confidence intervals of the estimated coefficients. Only those coefficients whose 95% confidence intervals do not overlap with zero are identified as significant connections. In the two examples displayed (Dataset-1), the cells numbered 3, 8, and 9 were classified as two FS neurons and a RS neuron, respectively. (a) Estimated GLM coefficients using complete (21 + 21) trials. (b) Estimated coefficients averaged from 20 Monte Carlo runs using only half of (11 + 11) trials (shaded bars show the standard error of the mean). (c) Estimated GLM coefficients averaged from 100 Monte Carlo runs using shuffled successful/unsuccessful trial ID.

However, this method also makes an assessment of how influential different, brief windows (3–10 ms) of the trigger neuron's firing history are on the firing of the target neuron. This allows for an observation of the dynamics of functional connections between two neurons at different latencies. When comparing the spiking associations between pairs of neurons during successful and unsuccessful task trials, it was observed that the spiking dependence that was inferred between the trigger neuron and the target neuron was quite different during different history windows, in terms of either being statistically significant nonzero or their excitatory/inhibitory effects. In some cases, the coefficient trace over history time lags would be completely reversed between trials of differing success (e.g., see the coefficient traces of two representative pairs of neurons illustrated in Figure 4(a)). The difference of their firing patterns is deemed to be statistically significant, as indicated by the 95% confidence bounds in the estimates). This suggests that the timing and dynamics of (some, if not all) neuronal interactions between task-related neurons have a role in leading to successful task performance. This phenomenon was observed in all subtype pairings and across all datasets. To test against the null hypotheses that the observed differences in spiking associations occurred due to overfitting of our model, or were simply observed by chance, we also carried out two follow-up procedures as sanity checks for our data. To test for overfitting, we randomly split the number of trials used in the analysis into half for both the successful and unsuccessful groups and recomputed the GLM coefficients. The procedure was repeated for 20 Monte Carlo runs, and the mean (and standard error of the mean, SEM) coefficients are shown in Figure 4(b). Comparing Figures 4(a) and 4(b), a high degree of positive correlation of the estimate can be observed, implying that model overfitting was not an issue in our analysis. In order to confirm that the observed differences did not occur due to chance, we randomly shuffled the trial IDs amongst the succ/unsucc groups (so that each group contained equal numbers of both successful and unsuccessful trials) and repeated the same analysis. The mean/SEM estimates of the coefficients are shown in Figure 4(c). Comparing Figures 4(a) and 4(c), it is obvious that the coefficients estimated from shuffled trials are not significantly different from zero, and the results with mixed task performance trials appear almost identical (Figure 4(c), between the upper and lower panels). This confirms that the differences observed in the spiking associations between the neuron pairs showed in Figure 4(a) did not occur by chance and were in fact related to the differences in the outcome of task performance. These findings indicate that while the general incidence of spiking associations between neuronal pairs may remain stable, more finely tuned temporal features of these associations may affect the outcome of task performance. At present, we must rely on visual inspection in order to identify differences in coefficient traces. Thus, it remains difficult (without subjective judgement) to quantify the degree to which two coefficient traces are different. For this reason, we do not yet have a summary statistic for the datasets analysed here. 

### 3.8. Changes in Spiking Associations Were Not Related to Changes in Single Unit Activity

Only task-related neurons were included in these analyses, and although it is beyond the scope of this particular study to discuss single unit activity modulation during task performance in detail, it is important to point out that there was no observable relationship between single unit activity and the variation seen in the spiking associations described in this study. Single neuron spiking modulation during task performance was studied during successful and unsuccessful task trials. In approximately 40% of cells (data not shown here), there was no significant difference in the timing or frequency of spiking rate modulation when successful trials were compared with unsuccessful trials. The remainder of cells showed differences between the two behavioural conditions that varied from slight temporal variances, to clear differences in spiking frequency. However, the observed differences in spiking associations were seen in similar proportions regardless of whether or not neurons showed observable differences in single unit responses between successful and unsuccessful trials.

## 4. Discussion

### 4.1. What Do Spiking Associations Tell Us?

This study utilized a point process GLM method in order to infer functional connectivity between cells that displayed significant levels of covariation in their spiking patterns. However, it is important to establish that in this particular study, only cells recorded from different microwires were considered for network analysis. Given the distance between recording sites (>1 mm), it is unlikely that detected functional connections were a result of direct anatomical connections. Further evidence to support this inference is the fact that associations detected between RS-RS pairs were often inhibitory and FS-FS pairings were often excitatory. Therefore the most probable explanation for these spiking associations is that functionally connected cells are receiving input from a common source in order to drive their discharge, a concept that has been proposed by studies in the past [5–7]. It has been well established that MI receives strong inputs from many cortical [46] and subcortical [47] sources that are involved in the planning and preparation of motor tasks. It is possible that inputs from these regions are responsible for the rapid increase in spiking associations that we see during reaching. Simultaneous recordings of other cortical motor areas with MI would be a worthwhile future experiment in order to confirm this hypothesis. 

### 4.2. More Spiking Associations Were Detected during Reaching Than Background

The majority of studies that identify covariation in spiking patterns amongst MI neurons exclusively investigate periods of task performance, while fewer studies have considered network properties during premovement periods [48]. The current study investigated the prevalence and nature of spiking associations that occurred both during the task and the period of time prior to movement onset (background period) in order to evaluate whether differences in movement accuracy were predictable in interactions occurring prior to movement onset. Spiking associations between neurons during task performance were more common than during background periods, as has been described in previous studies [7, 9]. The observed increases in spiking associations during task performance were seen in all cell subtype pairings, but most markedly in RS-RS pairs, suggesting that a larger proportion of pyramidal cells establish spiking associations with one another during the performance of a skilled movement. Despite the rapid increase in RS-RS associations seen during task performance, the findings of this study indicate that in MI, FS neurons were significantly more likely than RS neurons to establish spiking associations with other neurons. Increased prevalence of coincident spiking activity amongst FS neurons has been previously observed in both intracellular [49, 50] and extracellular [19, 51] studies in nonmotor areas of the cortex. During both background and reaching periods FS-FS couplings were proportionately the most commonly seen, followed by RS-FS and RS-RS, a finding similar to what has been described in layer V neurons in the prefrontal and somatosensory cortices [18, 52]. Interestingly, the outcome of task performance did not significantly affect the prevalence of detected spiking associations in either background or reaching periods, suggesting that regardless of trial success, similar networks of neurons are being recruited during performance of the reaching task. However, a novel aspect of our methodology allowed for the computation of the mean (*E* + *I*) connectivity ratio. The results of this calculation indicated that during successful trials, detected functional connections were significantly stronger and more consistent across multiple history windows than during unsuccessful trials. This suggests that the performance of inaccurate movement patterns may be a result of less robust transmission of the input that is reaching and driving the activities of MI neurons.

### 4.3. Balance of Excitation and Inhibition in MI

Previous studies have shown significant increases in the prevalence of spiking associations between background periods and periods of task performance [7, 9]. However, this is the first study to show that this finding is not limited to excitatory associations, but also involves a proportionately similar increase in inhibitory functional connections. Cortical neurons are known to receive a specific ratio of excitatory and inhibitory inputs in order to control their discharge frequency and timing [53]. This balance of inputs can be disrupted by a number of factors such as induction of anesthesia [54] or pathology [55, 56]. Since the methods used in this study allowed for the accurate detection of inhibitory connections as well as excitatory connections, we found that an animal shifting its behaviour from postural maintenance to target reaching is not a sufficient stimulus to disrupt the balance of excitatory to inhibitory interactions influencing the activities of MI neurons.

### 4.4. Trial Success Affects the Timing of Detected Spiking Associations

When a significant spiking association is detected between simultaneously recorded cells, this does not necessarily infer coincident spiking. Rather, the firing of a reference neuron may influence the firing of a target neuron many milliseconds later, introducing the concept of spiking associations at different latencies. Studies have shown that when neurons develop spiking associations, any latency in the detected association will remain stable to millisecond precision across many presentations of the same stimulus [57]. The present study shows that spiking associations occurring between the same neurons have significant differences in their temporal features depending on whether task performance is successful or unsuccessful. Thus, movement errors may occur due to abnormal neural interactions within MI which are probably related to abnormal inputs to MI neurons from other cortical and subcortical regions.

## 5. Conclusions

This is the first study to investigate the effect of unforced movement errors made during performance of a reaching task on the nature of functional connectivity between multiple neurons located in MI. The major findings of this work infer that many network interactions do not change between motor behaviours (e.g. ratios of excitatory and inhibitory interaction). However, errors in reaching are associated with significant disruption of the strength, duration, and timing of functional connections. This has implications for our understanding of the dynamic nature of the networks involved in motor learning in MI.

## Figures and Tables

**Table 1:** Summary of experimental data in the present study.

Dataset	Number of trials (succ/unsucc)	Number of neurons (RS/FS)	Forelimb/Hindlimb
		Animal 1	

1	42 (21/21)	13 (8/5)	7/6
2	50 (26/24)	15 (10/5)	9/6
3	31 (15/16)	15 (7/8)	10/5
4	35 (16/19)	11 (5/5)	6/4

		Animal 2	

5	37 (26/11)	10 (6/4)	6/4
6	60 (32/28)	10 (7/3)	7/3
7	42 (27/15)	9 (8/1)	6/3
8	35 (24/11)	12 (6/6)	6/6

**Table 2:** Mean duration ± SE (ms) at each stage of task performance across trials for two animals during successful and unsuccessful trials.

	Number of trials	Duration (ms)		
		Premovement	Reach	Withdraw
		Animal 1		

Succ.	78	136 ± 11	317 ± 15	507 ± 14
Unsucc.	80	131 ± 13	372 ± 18	475 ± 17

		Animal 2		

Succ.	109	156 ± 16	606 ± 11	812 ± 24
Unsucc.	65	162 ± 12	682 ± 14	772 ± 21

**Table 3:** The mean (*E* + *I*) connectivity ratio and the number of inferred significant neuronal associations (including self-interaction) in three cell-type subcategories in 8 datasets. The first two numbers in each column show the prevalence of connected neuronal pairs as a fraction of the total number of possible connections during unsuccessful and successful (in brackets) trials, respectively. The numbers in square brackets in the last 3 columns indicate the numbers of all possible pairwise neuronal associations (including self-interaction) for different data sets. Two neurons are said to interact if their pairwise connection coefficients at any time lag are significantly different from 0. Note that in order to compute the fraction of significant neuronal interactions in the three cell-pair subcategories, we need to divide the listed raw numbers by the respective numbers in the square brackets in each group.

Dataset	Condition	Mean (*E* + *I*) ratio	Ratio of significant neuronal interactions		
			RS-RS	RS-FS	FS-FS
1	Background	0.08 (0.07)	0.3 (0.28) [64]	0.38 (0.35) [80]	0.88 (0.88) [25]
	reaching	0.09 (0.12)	0.61 (0.63)	0.4 (0.41)	0.96 (1)
2	Background	0.45 (0.46)	0.27 (0.29) [100]	0.24 (0.24) [100]	0.6 (0.64) [25]
	reaching	0.49 (0.61)	0.6 (0.64)	0.24 (0.27)	0.68 (0.68)
3	Background	0.26 (0.24)	0.02 (0.02) [49]	0.05 (0.08) [112]	0.25 (0.3) [64]
	reaching	0.29 (0.47)	0.31 (0.35)	0.2 (0.2)	0.47 (0.42)
4	Background	0.52 (0.49)	0.32 (0.24) [25]	0.24 (0.2) [50]	0.8 (0.76) [25]
	reaching	0.59 (0.72)	0.6 (0.64)	0.44 (0.44)	1 (1)

5	Background	0.56 (0.74)	0.19 (0.44) [36]	0.29 (0.44) [48]	0.81 (1) [16]
	reaching	0.62 (0.79)	0.36 (0.64)	0.25 (0.44)	0.81 (0.94)
6	Background	0.48 (0.53)	0.18 (0.2) [49]	0.19 (0.24) [42]	0.78 (0.89) [9]
	reaching	0.56 (0.86)	0.77 (0.77)	0.5 (0.5)	1 (1)
7	Background	0.14 (0.27)	0.03 (0.05) [64]	0.13 (0.31) [16]	1 (1) [1]
	reaching	0.19 (0.48)	0.2 (0.3)	0.19 (0.25)	1 (1)
8	Background	0.14 (0.27)	0 (0.08) [36]	0.08 (0.18) [72]	0.69 (0.92) [36]
	reaching	0.16 (0.26)	0.03 (0.08)	0.1 (0.25)	0.81 (0.89)

**Table 4:** Summary of multiway ANOVA.

Source	Sum Sq.	d.f.	Mean Sq.	*F*	Prob > *F*
Cell group (*g*1)	3.7087	2	1.8543	54.29	1*e* − 8
Task outcome (*g*2)	0.0506	1	0.0506	1.48	0.2270
Period (*g*3)	0.5797	1	0.5797	16.97	1*e* − 4
Error	2.6981	79	0.0342		

Total	7.0371	83			
